# Phenotype Scoring and Personalized Analysis of Population‐Scale Single‐Cell Data Dissects the Complexity of Alzheimer's Disease

**DOI:** 10.1002/alz70855_097917

**Published:** 2025-12-23

**Authors:** Daifeng Wang

**Affiliations:** ^1^ University of Wisconsin ‐ Madison, Madison, WI, USA

## Abstract

**Background:**

The complexity of Alzheimer's disease (AD) manifests in diverse clinical phenotypes, including cognitive impairment and neuropsychiatric symptoms (NPSs). Such heterogeneity can be attributed to the variations in cellular and molecular mechanisms across individuals, especially at the single‐cell level. However, the etiology of these phenotypes remains elusive. To address this, the PsychAD project generated a population‐level single‐nucleus RNA‐seq dataset comprising over 6 million nuclei from the prefrontal cortex of 1,494 individual brains, covering a variety of AD‐related phenotypes that capture cognitive impairment, severity of pathological lesions, and the presence of NPSs.

**Method:**

Using emerging deep learning approaches, we analyzed the PsychAD data to score single‐cell phenotype associations and identified phenotype associate cells (PACs). We also analyzed personalized single‐cell functional genomics involving cell type interactions and gene regulatory networks. In particular, we learned latent representations of individual functional genomics (embeddings) and quantified importance scores of cell types, genes, and their interactions for each individual. Finally, we associated genetic variants with the changes of cell type‐gene regulatory networks across individuals, i.e., gene regulatory QTLs (grQTLs), providing novel functional genomic insights compared to existing QTLs.

**Result:**

We identified ∼1.5 million PACs and compared them across 27 distinct brain cell subclasses, prioritizing cell subpopulations and their expressed genes across various AD phenotypes. This includes the upregulation of a reactive astrocyte subtype with neuroprotective function in AD resilient donors. Additionally, we identified PACs that link multiple phenotypes, including a subpopulation of protoplasmic astrocytes that alter their gene expression and regulation in AD donors with depression. Moreover, our embeddings improved phenotype classifications and revealed potentially novel subtypes and population trajectories for AD progression, cognitive impairment, and NPSs. Our importance scores prioritized personalized functional genomic information and showed significant differences in cell‐type regulatory mechanisms. Such information also allowed us to further identify subpopulation‐level biological pathways such as ancestries.

**Conclusion:**

Overall, our analyses have advanced in uncovering the cellular and molecular mechanisms underlying diverse AD phenotypes, which has the potential to provide valuable insights for identifying novel diagnostic markers and therapeutic targets. Our results are available through open‐source web apps, as well as a personalized single‐cell functional genomic atlas for AD.

